# Tracking the shift in enteric fever trends and evolving antibiotic sensitivity patterns

**DOI:** 10.4314/gmj.v58i1.12

**Published:** 2024-03

**Authors:** Yoganathan Chidambaram, Clement J Dhas, R Juhi, Velammal Petchiappan, S Sujithkumar

**Affiliations:** 1 Department of General Medicine, PSG Institute of Medical Sciences and Research, Coimbatore, Tamil Nadu, India

**Keywords:** Salmonella Typhi, Salmonella Paratyphi_A, Enteric fever, Antibiotic Sensitivity, Retrospective study

## Abstract

**Objective:**

This study aims to examine the frequency of *Salmonella* Paratyphi found in blood cultures and evaluate the antibiotic susceptibility pattern of *Salmonella* isolates to different antibiotics. Additionally, the study aims to assess the paradigm shift in the trend of enteric fever caused by *Salmonella* Typhi (*S*. Typhi) to *Salmonella* Paratyphi(*S*. Paratyphi) .

**Study Design:**

Retrospective study

**Participant:**

The study enrolled patients aged 12 years and above diagnosed with enteric fever (positive blood culture) and admitted to Peelamedu Samanaidu Govindasamy Naidu (PSG) Hospital.

**Interventions:**

The study analyzed demographic and antibiotic susceptibility profiles of *Salmonella* isolates collected from 106 enteric fever patients in the hospital between 2010 and 2022. The susceptibility profiles of *Salmonella* isolates to multiple antibiotics were assessed.

**Results:**

There were 106 participants, and 95 (89.62%) of them had enteric fever linked to *Salmonella* Typhi, while only 11 (10.38%) had enteric fever linked to *Salmonella* Paratyphi A. From 2010 to 2022, the study discovered a general decline in the prevalence of enteric fever caused by *Salmonella* species. But between 2014 and 2022, the incidence of enteric fever linked to *S. Typhi* rapidly increased. Azithromycin (100% , n = 106) and ceftriaxone (99% , n = 105) were highly effective against the *Salmonella* isolates, whereas nalidixic acid was resisted by 3 isolates (4.72%, n = 3).

**Conclusion:**

The study observed a higher incidence of *Salmonella* Typhi in comparison to Paratyphi A and a greater susceptibility of males to enteric fever.

**Funding:**

None declared

## Introduction

Gram-negative bacteria, notably *S.* Typhi (typhoid fever) and *S*. Paratyphi (paratyphoid fever) are the source of enteric fever, an infectious disease that can be caught by tainted food and water. Poor hygiene habits, poor sanitation, low socioeconomic level, and consuming food or water contaminated with faeces from either acutely infected individuals or chronic, asymptomatic carriers are the key risk factors for this illness.^1^,^2^

Earlier research has established enteric fever as a crucial public health concern worldwide, with yearly records of more than 21.6 million cases and no less than 250,000 fatalities. Previous studies have shown that enteric fever is a serious public health issue that affects the entire world, with annual records of more than 21.6 million cases and at least 250,000 fatalities. In underdeveloped countries like India, the disease prevalence ranges from 102 to 2,219 per 100,000 people. In Nigeria, typhoid fever remains a significant issue due to urbanization, limited clean water, immigrant worker mobility, poor waste management, strained healthcare, and antibiotic overuse fostering antibiotic-resistant *S*. Typhi. Yet, research indicates varying prevalence, from 0.071% in Oyo to 47.1% in Osun.^3^,^4^,^5^,^6^

Systemic febrile sickness typhoid fever requires prompt antibiotic treatment. In the past few decades, Typhi bacteria have become increasingly resistant to first-line antibiotics such as chloramphenicol, ampicillin, and trimethoprim-sulfamethoxazole, leading to the use of fluoroquinolones like ciprofloxacin as the primary treatment option. However, since the early 2000s, there has been a surge in fluoroquinolone non-susceptibility, including intermediate or complete resistance to ciprofloxacin, particularly in South Asia.

As a result, third-generation cephalosporins, such as ceftriaxone, are now recommended as the new first-line treatment. In Pakistan, a typhoid fever outbreak caused by Salmonella enterica serotype Typhi occurred in February 2018. The bacteria were resistant to several antibiotics, such as ampicillin, chloramphenicol, fluoroquinolones, trimethoprim-sulfamethoxazole, and third-generation cephalosporins. The emergence of fluoroquinolone non-susceptible strains resistant to third-generation cephalosporins like ceftriaxone further complicated this outbreak. As a result, typhoid illness has become more difficult worldwide and not only in Pakistan.^7^,^8^,^9^

The rise of the novel, extensively drug-resistant (XDR) strains of *S*. Typhi is alarming, with reports of them appearing for the first time in Pakistan. These strains have expanded globally, including in the USA, and are resistant to first-line medications such as ciprofloxacin and ceftriaxone.^10^,^11^ Typhoid fever treatment costs, mortality, and morbidity have all increased as a result of the rise in antibiotic resistance. However, these pricey carbapenems and azithromycin, which continue to be the backbone of treatment for infected patients, are effective against these XDR strains of *Salmonella*.^12^,^13^,^14^,^15^

The ever-evolving antimicrobial resistance patterns of *Salmonella* Typhi in typhoid fever necessitate frequent studies to identify new resistant strains in different regions on time. Our investigation seeks to evaluate the incidence and antibiotic sensitivity patterns of *Salmonella* isolates collected from patients with enteric fever.

## Methods

### Study population

A retrospective analysis was carried out with patients admitted to Peelamedu Samanaidu Govindasamy Naidu (PSG) Hospital between January 2010 and December 2022. The study comprised patients over 12 years old who were admitted to PSG Hospital with enteric fever (positive Blood culture). Case records of about 132 patients with enteric fever were scrutinized. Among them, 26 were excluded because of the lack of blood culture (a clinical diagnosis of enteric fever was made).

### Study design and methodology

Between 2010 and 2016, conventional blood culture bottles were used to culture all collected blood samples, while the BACTEC blood culture system was adopted from 2017 onwards for the purpose of *Salmonella* isolatio*n*. The Salmonella serotype was identified through the triple sugar iron test (based on H_2_S production) from 2010 to 2018 and the VITEK method from 2019 onwards. Investigation of the trend of *Salmonella* species causing enteric fever between 2010 and 2022, as well as the susceptibility of identified serotypes to a range of antibiotics, including trimethoprim + sulfamethoxazole, ceftriaxone, nalidixic acid, fluoroquinolones, azithromycin, ampicillin, and chloramphenicol was also performed. Data were entered in Excel, and statistical analysis was done in SPSS version 28. The frequency and percentage were used to explain the categorical variables, whereas the continuous variables were presented in terms of mean, standard deviation, minimum, and maximum values.

### Ethical clearance

Ethics approval was obtained from Institutional Human Ethics Committee (IHEC), PSG IMS&R (Ref. No. PSG/IHEC/2023/Appr/Exp/006) on January 05, 2023

## Results

**Figure 1:**
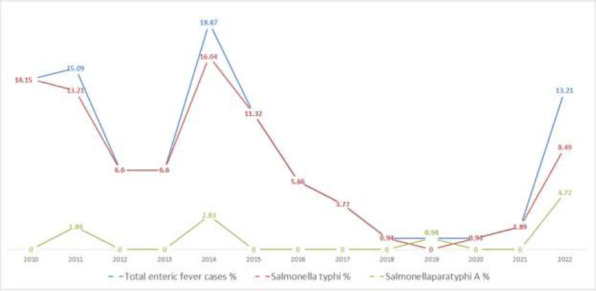
Trend of *Salmonella* species causing enteric fever over a period between 2010 to 2022

Among 106 participants, incidences of *S*. Typhi-associated enteric fever were observed in 95 (89.62%) participants, whereas only 11(10.38%) participants showed *S*. Paratyphi A associated enteric fever with a male preponderance (80 males and 26 females). The study showed a decreased rate of total enteric fever cases from 2011 (15.09 %) to 2013 (6.60%) and from 2014 (18.87%) to 2020 (0.94%). However, there was a significant increase in the overall number of cases of enteric fever between 2013 (6.60%) and 2014 (18.87%) and 2021 (1.89%) and 2022 (13.21%). *S*. Typhi and *S*. Paratyphi infections escalated from 2013 (6.60% and 0.0%) to 2014 (16.04% and 2.83%, respectively), and from 2021 (1.89% and 0.0%) to 2022 (8.49% and 4.72%, respectively). From 2011 (13.21% and 1.89%, respectively) to 2013 (6.60% and 0.0%, respectively) and from 2014 (16.04% and 2.83%, respectively) to 2021 (1.89% and 0.0%, respectively), there was a decline in the infection rate for both *S*. Typhi and *S*. Paratyphi infections (Figure 1).

The sensitivity of the Salmonella isolates (S. Typhi and Paratyphi) to various antibiotics was evaluated. Table 1 compiles the antimicrobial susceptibility profile of all isolates to the seven antimicrobial drugs. Both strains of Salmonella exhibited perfect resistance to nalidixic acid (95.28%) and the highest sensitivity to azithromycin (100%) followed by ceftriaxone (99%).

**Table 1:** Antibiotic Sensitivity Pattern of *Salmonella* species

Antibiotics	Sensitivity	n (%)
**Tmp + SM**	**Sensitive**	**101 (95.28)**
	**Resistant**	**5 (4.72)**
**Ceftriaxone**	**Sensitive**	**105 (99.06)**
	**Resistant**	**1(0.94)**
**Nalidixic acid**	**Sensitive**	**5(4.72)**
	**Resistant**	**101 (95.28)**
**Fluoroquinolones**	**Sensitive**	**67 (63.21)**
	**Resistant**	**39 (36.79)**
**Azithromycin**	**Sensitive**	**106 (100.00)**
	**Resistant**	**0 (0.00)**
**Ampicillin**	**Sensitive**	**95 (89.62)**
	**Resistant**	**11 (10.38)**
**Chloramphenicol**	**Sensitive**	**101 (95.28)**
	**Resistant**	**5 (4.72)**

*Note*: n-number of participants with enteric fever, %-the percentage of participants with enteric fever, Tmp + SM - Trimethoprim + sulfamethoxazole

All patients were discharged after an afebrile period of at least 48 hours

## Discussion

The current study concentrated on the presence of *S*. Typhi and *S*. Paratyphi in blood cultures obtained from patients with enteric fever and their susceptibility to various antimicrobial drug classes, such as combination of trimethoprim and sulfamethoxazole, ceftriaxone, nalidixic acid, fluoroquinolones, azithromycin, ampicillin, and chloramphenicol.

In this study, we observed that *S*. Typhi infection was prevalent in most patients (89.62%) diagnosed with enteric fever, with men being the more commonly affected gender. The higher incidence of enteric fever among men could be attributed to variations in *S*. Typhi exposure, potentially linked to gender-related differences in dietary practices and hygiene habits. Additionally, the male predominance in typhoid fever might be influenced by differences in the inflammatory response patterns within host Peyer's patches triggered by *S*. Typhi or the natural exposure of Peyer's patches to *S*. Typhi, considering sexual dimorphism. These findings align with similar results reported by Umair and Siddiqui, 2020, where *S*. Typhi was isolated in 79.5% of cases and *S*. Paratyphi in 20.5%, with a male-to-female ratio of nearly 2:1.

Among these cases, males constituted 66.3%, while females accounted for 33.7%.^16^,^17^,^18^

The 12-year analysis of the trajectory of enteric fever cases caused by *Salmonella* species revealed a sharp increase in cases from 2013 to 2014 (12.27%) and from 2021 to 2022 (11.32%). Furthermore, in cases where the isolated strains are nalidixic acid-resistant, the majority of Salmonella isolates exhibited sensitivity to azithromycin, ceftriaxone, and chloramphenicol. One of the primary causes of quinolone resistance is thought to include mutations in the quinolone resistance-determining region (QRDR) of *gyrA*, a subunit of the DNA gyrase enzyme.^19^ Another explanation may include transferable plasmid-mediated quinolone resistance (PMQR) genes such as pentapeptide repeat protein-encoding *qnrA, qnrB*, and *qnrS*; an efflux-pump-encoding *qepA*; and an aminoglycoside acetyltransferase-encoding enzyme variant *aac(6′)-Ib-cr* which may cause low-level fluoroquinolones resistance.^20^,^21^,^22^,^23^ In instances of *S*. Pratyphi in India, as noted in the study by Sharvani *et al.*, 2016 the presence of Nalidixic Acid Resistance (NAR) signifies a form of reduced resistance to ciprofloxacin, potentially leading to treatment ineffectiveness.

Strains that are initially resistant to Nalidixic Acid may necessitate fewer exposures to fluoroquinolones (FQ) to develop strong resistance to ciprofloxacin compared to strains that are initially susceptible to ciprofloxacin.^20^,^24^,^25^,^26^ In another study conducted by Adabara, *et al.*, 2012 in a Military Hospital in Minna, Nigeria, it was found that *S*. Typhi isolates exhibited resistance to key antibiotics commonly prescribed in the study region for treating typhoid fever, including ceftriaxone, cefuroxime, amoxicillin, ampicillin, ciprofloxacin, and combination of amoxicillin and clavulanic acid (augmentin).

Interestingly, they showed sensitivity to chloramphenicol and ofloxacin, but these antibiotics were not typically utilized for typhoid fever treatment in that area. The study identified the presence of multiple drug-resistant (MDR) strains of S. Typhi, which could be attributed to the overuse of a limited selection of antibiotics and potential issues with an accurate diagnosis, leading to the emergence and dissemination of resistant *S*. Typhi strains. Consequently, the study underscores the importance of a collaborative effort between physicians and laboratories when selecting antibiotics for bacterial disease treatment, aiming to curb the development of antibiotic-resistant bacterial pathogens.^27^

There is a trend of changes in enteric fever from *S*. Typhi to *S*. Paratyphi over the years. Zellweger *et al.*, 2017 have discussed the possible reasons behind this as various environmental, ecological, or epidemiological factors.^28^ Another explanation is that there are currently two vaccines available for the protection of typhoid fever. The parenteral Vi vaccine is based on the *S*. Typhi Vi antigen, whereas the live attenuated oral Ty21a vaccine contains the *S*. Typhi strain Ty21a. Since *S*. Paratyphi A lacks the Vi- polysaccharide capsule antigen, Vi-based vaccines are unlikely to protect against paratyphoid fever.^29^

The retrospective approach of the current study, which relies on previously gathered data, may be subjected to information and selection biases, imposing constraints on its scope. The research was restricted to a single centre, further limiting the generalizability of the results to other settings. Additionally, the absence of follow-up details hinders the assessment of long-term outcomes and the establishment of causal relationships. Future research should address these limitations to provide a more comprehensive understanding of the topic. Another drawback of the study is that the vaccination status of the study participants was not included.

## Conclusion

In this study, *S*. Typhi was the most common cause of enteric fever, and it mostly affected men. Both Salmonella strains showed susceptibility against traditional first-line antibiotics but resistance to quinolone antibiotics such as nalidixic acid. It is also suggested that to treat salmonellosis cases caused by ciprofloxacin (fluoroquinolones) resistant Salmonella strains, a third-generation cephalosporin, such as cefotaxime or ceftriaxone, is the first choice.

However, if resistance to these antibiotics develops, the available treatment options will be carbapenems or fourth generation cephalosporins, tigecycline, or a combination antibiotic therapy. In view of the constantly shifting environment, this study emphasizes the necessity for ongoing evaluation and prudent antimicrobial use. For the primary prevention of illness, public health awareness efforts and immunization programs against Salmonella should also be taken into consideration.

## References

[1] Patil N, Mule P (2019). Sensitivity Pattern Of Salmonella typhi And Paratyphi A Isolates To Chloramphenicol And Other Anti-Typhoid Drugs: An In Vitro Study. Infect Drug Resist.

[2] Mogasale V, Maskery B, Ochiai RL, Lee JS, Mogasale VV, Ramani E, Kim YE, Park JK, Wierzba TF (2014). Burden of typhoid fever in low-income and middle-income countries: a systematic, literature-based update with risk- factor adjustment. Lancet Glob Health.

[3] Zaki SA, Karande S (2011). Multidrug-resistant typhoid fever: a review. J Infect Dev Ctries.

[4] Crump JA, Luby SP, Mintz ED (2004). The global burden of typhoid fever. Bull World Health Organ.

[5] Kothari A, Pruthi A, Chugh TD (2008). The burden of enteric fever. J Infect Dev Ctries.

[6] Akinyemi KO, Oyefolu AOB, Mutiu WB (2018). Typhoid Fever: Tracking the Trend in Nigeria. Am J Trop Med Hyg.

[7] Chatham-Stephens K, Medalla F, Hughes M (2019). Emergence of Extensively Drug-Resistant Salmonella Typhi Infections Among Travelers to or from Pakistan - United States, 2016-2018. MMWR Morb Mortal Wkly Rep.

[8] Centre for Disease Control and Prevention (2022). Symptoms and Treatment.

[9] Crump JA, Sjölund-Karlsson M, Gordon MA, Parry CM (2015). Epidemiology, Clinical Presentation, Laboratory Diagnosis, Antimicrobial Resistance, and Antimicrobial Management of Invasive Salmonella Infections. Clin Microbiol Rev.

[10] Su LH, Wu TL, Chia JH, Chu C, Kuo AJ, Chiu CH (2005). Increasing ceftriaxone resistance in Salmonella isolates from a university hospital in Taiwan. J Antimicrob Chemother.

[11] González-López JJ, Piedra-Carrasco N, Salvador F (2014). ESBL-producing Salmonella enterica serovar Typhi in traveler returning from Guatemala to Spain. Emerg Infect Dis.

[12] Ali Shah SA, Nadeem M, Syed SA, Fatima Abidi ST, Khan N, Bano N (2020). Antimicrobial Sensitivity Pattern of Salmonella Typhi: Emergence of Resistant Strains. Cureus.

[13] Buckle GC, Walker CL, Black RE (2012). Typhoid fever and paratyphoid fever: Systematic review to estimate global morbidity and mortality for 2010. J Glob Health.

[14] Qamar FN, Azmatullah A, Bhutta ZA (2015). Challenges in measuring complications and death due to invasive Salmonella infections. Vaccine.

[15] Ochiai RL, Acosta CJ, Danovaro-Holliday MC, Domi Typhoid Study Group (2008). A study of typhoid fever in five Asian countries: disease burden and implications for controls. Bull World Health Organ.

[16] Khan M (2012). A plausible explanation for male dominance in typhoid ileal perforation. Clin Exp Gastroenterol.

[17] Kanungo S, Dutta S, Sur D (2008). Epidemiology of typhoid and paratyphoid fever in India. J Infect Dev Ctries.

[18] Umair M, Siddiqui SA (2020). Antibiotic Susceptibility Patterns of Salmonella Typhi and Salmonella Paratyphi in a Tertiary Care Hospital in Islamabad. Cureus.

[19] Ryan MP, Dillon C, Adley CC (2011). Nalidixic acid-resistant strains of Salmonella showing decreased susceptibility to fluoroquinolones in the midwestern region of the Republic of Ireland due to mutations in the gyrA gene. J Clin Microbiol.

[20] Song Q, Xu Z, Gao H, Zhang D (2018). Overview of the development of quinolone resistance in Salmonella species in China, 2005-2016. Infect Drug Resist.

[21] Fàbrega A, Madurga S, Giralt E, Vila J (2009). Mechanism of action of and resistance to quinolones. Microb Biotechnol.

[22] Giraud E, Baucheron S, Cloeckaert A (2006). Resistance to fluoroquinolones in Salmonella: emerging mechanisms and resistance prevention strategies. Microbes Infect.

[23] Hopkins KL, Davies RH, Threlfall EJ (2005). Mechanisms of quinolone resistance in Escherichia coli and Salmonella: recent developments. Int J Antimicrob Agents.

[24] Sharvani R, Hemavathi, Dayanand DK, Shenoy P, Sarmah P (2016). Antibiogram of Salmonella Isolates: Time to Consider Antibiotic Salvage. J Clin Diagn Res.

[25] Muthu G, Suresh A, Sumathy G, Srivani R (2011). Studies on antimicrobial susceptibility pattern of salmonella isolates from Chennai, India. Int J Pharm Bio Sci.

[26] Jain S, Chugh TD (2013). Antimicrobial resistance among blood culture isolates of Salmonella enteric in New Delhi. J Infect Dev Ctries.

[27] Adabara NU, Ezugwu BU, Momojimoh A, Madzu A, Hashiimu Z, Damisa D (2012). The Prevalence and Antibiotic Susceptibility Pattern of Salmonella typhi among Patients Attending a Military Hospital in Minna, Nigeria. Adv Prev Med.

[28] Zellweger RM, Basnyat B, Shrestha P (2017). A 23-year retrospective investigation of Salmonella Typhi and Salmonella Paratyphi isolated in a tertiary Kathmandu hospital. PLoS Negl Trop Dis.

[29] Crump JA, Mintz ED (2010). Global trends in typhoid and paratyphoid Fever. Clin Infect Dis.

